# Identifying breast cancer patients who gain the most dosimetric benefit from deep inspiration breath hold radiotherapy

**DOI:** 10.1002/jmrs.415

**Published:** 2020-07-05

**Authors:** Patricia Browne, Nakia‐Rae Beaton, Harish Sharma, Sharon Watson, G Tao Mai, Jennifer Harvey, Anne Bernard, Elizabeth Brown, Catriona Hargrave, Margot Lehman

**Affiliations:** ^1^ Radiation Oncology Department Cancer Services Princess Alexandra Hospital Brisbane Queensland Australia; ^2^ School of Medicine University of Queensland Brisbane Queensland Australia; ^3^ QFAB Bioinformatics Institute for Molecular Bioscience University of Queensland Brisbane Queensland Australia; ^4^ School of Clinical Sciences Queensland University of Technology Brisbane Queensland Australia

**Keywords:** breast radiation therapy, deep inspiration breath hold, heart dose, potential factors

## Abstract

**Introduction:**

Deep inspiration breath hold (DIBH) has been proven to reduce cardiac dose for women receiving left breast and chest wall radiation therapy. However, it utilises extra departmental resources and patient exertion. The aim of this exploratory study was to investigate if any factors existed that could identify breast cancer patients who may benefit most from DIBH, to facilitate appropriate utilisation of departmental resources.

**Methods:**

Left‐sided breast cancer patients aged 18–70 years, and right‐sided breast cancer patients with internal mammary nodes included, were recruited. DIBH and free breathing (FB) plans were created for all patients. Patient demographic and clinical history were recorded. Variables including lung threshold value, lung volume, patient separation, maximum heart in field, volume of planning target volume (PTV), heart dose, ipsilateral lung dose were compared between plans.

**Results:**

Plans for 31 patients were analysed. No correlations were found between lung threshold value or patient separation and cardiac dose. Moderate to strong correlations were found with BMI, PTV volume and lung volume change however no definitive thresholds were determined. A significant difference was found in the maximum heart in field between DIBH and FB (*P* < 0.001) with those patients with greater than 0.7 cm heart in the field on the FB scan demonstrating greater reductions in mean heart dose.

**Conclusion:**

Maximum heart in the field of greater than 0.7 cm in FB could be a potential factor to identify patients who may benefit most from DIBH. This factor warrants investigation in a larger patient cohort to test its validity.

## Graphical abstract

The aim of this study was to investigate factors that could help identify breast cancer patients who may benefit most from DIBH, to facilitate appropriate utilisation of departmental resources. Only factors that could be evaluated after CT simulation were found to have a significant relationship with reduction in heart dose. The only possible threshold identified was the maximum heart in field in free breathing, with those patients with >0.7 cm heart in field experiencing greater reductions in mean heart dose.

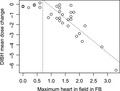

## Introduction

Breast cancer is the most common malignancy occurring in Australian women, with 1 in 7 women expected to be diagnosed with this disease.^1^ Surgery followed by radiation therapy has been shown to significantly reduce local recurrence and improve long term survival, however it has also been demonstrated that left‐sided breast or chest wall irradiation is associated with an increased risk of cardiac mortality.^2^, ^3^ Darby et al’s^3^ 2005 study showed that cardiac mortality ratios decreased for women treated with radiation therapy progressively from 1973 to 2001, concluding that the evolution of radiation therapy planning techniques may have been a contributing factor. Given the lack of follow up data after 10 years for women treated between 1993 and 2001, Darby et al’s^4^ 2013 study which included women treated up to 2001, found that each 1Gy increase in mean heart radiation dose was associated with a 7.4% increase in coronary events. There was also a linear, no‐threshold relationship between mean heart dose and the risk of subsequent major coronary events.^4^

Deep inspiration breath hold (DIBH) radiation therapy is a treatment technique that moves the heart inferiorly and away from the chest wall. It was introduced to facilitate a reduction in heart dose and potentially minimise associated cardiac toxicity. Although the success of DIBH for breast cancer patients to reduce cardiac dose is widely published, Latty et al’s^5^ 2015 review found there is limited data on reliable parameters for appropriately targeting those patients that will benefit most from DIBH treatment. DIBH techniques can be resource intensive on departments requiring both additional use of equipment and staff time together with the need to acquire and service new equipment.^6^ Increased patient exertion is also required as performing breath hold each day over a number of weeks may further add to radiation therapy related fatigue.^7^ It is also important to note that not all patients are able to meet the minimum breath hold times required during treatment field delivery or receive the same level of cardiac dose reduction.

To date, various potential patient and radiation therapy technique‐specific factors have been investigated as potential predictors of mean heart dose reduction.^6^, ^8^, ^9^, ^10^, ^11^, ^12^, ^13^, ^14^, ^15^, ^16^, ^17^, ^18^ While these studies have all compared heart doses on free breathing (FB) versus DIBH planning computed tomography (CT) scans of left‐sided breast cancer patients receiving radiation therapy to their whole breast or chest wall, predictive factors identified have varied. The aim of this study was to investigate if any factors existed that could identify breast cancer patients who may benefit most from DIBH, to develop a cardiac dose risk profile approach to facilitate appropriate utilisation of departmental resources.

## Methods

### Patient selection

Left‐sided breast cancer patients between the ages of 18–70 with any heart in the treatment field on a FB scan, and right‐sided breast cancer patients whose treatment involved internal mammary nodes, were recruited to this study. Patients were excluded if they were non‐English speaking and required a translator. This study was approved by Metro South Human Research Ethics Committee (HREC). Informed consent was obtained for all study participants.

### CT simulation

All potential patients were initially coached in DIBH to assess their suitability before progressing to CT planning. DIBH was achieved using the Active Breathing Coordinator (ABC) device (Elekta, Stockholm, Sweden). This device assists patients to hold their breath after deep inspiration at a lung volume (LV) which is comfortable for each patient. Each individual patient’s LV was determined during a DIBH coaching session conducted prior to CT simulation. The coaching session was also used to determine if patients were able to proceed with DIBH treatment. Patients needed to meet the following criteria: (1) be able to follow prompts from staff, (2) be able to establish a reproducible breathing pattern in regular breathing and (3) be able to hold their breath for a minimum of 20 sec. If patients were able to satisfy these criteria they underwent a FB and DIBH planning CT scan. All patients were positioned supine with arms up on a Posifix breast board (CIVCO, Iowa, USA) and customised T‐shaped VacQfix cushion (Qfix, Avandale, USA) under the shoulder region. A bolster was placed under the knees for comfort. Patients were scanned with 2 mm slice spacing on a Aquilion LB CT scanner (Toshiba Medical, Tochigi, Japan).

### Treatment planning

All patients were planned with a three‐dimensional conformal radiation therapy (3DCRT) treatment technique utilising tangential beams. All plans were generated in the Pinnacle^3^ treatment planning system (Philips Healthcare, Fitchburg, WI, USA). The breast or chest wall clinical tumour volume (CTV), planning target volume (PTV), heart and left anterior descending artery (LAD) contours were delineated by the radiation oncologist (RO). The planning radiation therapist (RT) contoured all other organs at risk (OARs). The clinical DIBH plan was completed first with a FB plan being generated retrospectively by a RT member of the study team. The prescribed dose was either 50 Gy in 25 fractions or 42.5Gy in 16 fractions. All DIBH plans met the departmental planning goals (Table 1) and routine quality assurance procedures. Each FB plan was optimised to achieve the lowest OAR dose while maintaining the required PTV coverage and was evaluated by an RT on the study team.

**Table 1:** Breast and chest wall DIBH planning goals

Structure	Goal	
CTV	V95%_Reference dose_	>98%
PTV	V95%_Reference dose_	>95%
Heart	V25Gy	<5%
	Mean	<3Gy
Ipsilateral lung	V5Gy	<40%
	V10	<35%
	V20	<15%
LAD	2cc	≤45Gy

CTV, Clinical target volume; LAD, Left anterior descending artery; PTV, Planning target volume.

### Data acquisition

Patient demographics and clinical history were recorded, including age, body mass index (BMI), primary site, stage, smoking status, co‐morbidities and radiation prescription. DIBH technique‐specific factors investigated in previous studies as potential factors associated with reduction in heart and lung dose were also collected,^6^, ^8^, ^9^, ^10^, ^11^, ^12^, ^13^, ^14^, ^15^, ^16^, ^17^, ^18^ and are provided in Table 2. Multiple heart, lung and LAD doses were recorded from both the FB and DIBH plans (Table 2). This was done to facilitate the analysis of various dose level reductions between the FB and DIBH plans with respect to each of the patient and technique‐specific factors collected.

**Table 2:** Variables collected for analysis

Variables collected	Comment
Lung threshold value (L)	Obtained from ABC device when the patient is in breath hold
Lung volume (cc)*	
Patient separation (cm)*	Measured as the distance between the medial and lateral entry points as chosen by the RO at simulation.
Maximum heart in field (cm)*	Measured as the point where the largest amount of heart was visible in the axial plane from the posterior edge of the treatment fields.
PTV volume (cc)*	
PTV dose*	V95%_Reference dose_
Heart dose*	Mean, maximum point dose, maximum dose to 1 cm^3^, V5Gy, V20Gy, V30Gy, V40Gy
Ipsilateral lung dose*	Mean, V5Gy, V20Gy, V30Gy
LAD dose*	Mean, maximum point dose, maximum dose to 1cm^3^, V5Gy, V20Gy, V30Gy, V40Gy

* Measured on both the DIBH and FB plans.

### Data analysis

The difference between variables measured on both the FB and DIBH plans were calculated. Descriptive statistics of each of the FB, DIBH and difference between FB and DIBH variables was performed. The mean and standard deviation (SD) was presented for continuous data that were normally distributed, and medians and inter‐quartile range (IQR) reported for continuous data that was not normally distributed. Normality was assessed using the Shapiro‐Wilk test. Categorical data were described using frequencies and percentages.

The patient ABC device lung threshold value was compared between smokers and non‐smokers using a two‐sample t‐test. The amount of heart in field for the FB and DIBH plans were compared using the Wilcoxon signed‐rank test.

Correlation analysis of the variables listed in Table 2 was performed using Pearson correlation (or Spearman’s rank correlation where appropriate) to determine the relationship between.

- The ABC device lung threshold value and age as well as DIBH dose change in the heart LAD and ipsilateral lung.
- FB patient separation and DIBH dose change in the heart, LAD and ipsilateral lung.
- BMI and dose changes in the heart, LAD and ipsilateral lung between FB and DIBH.
- PTV coverage and DIBH heart dose change.
- DIBH percentage change in lung volume and DIBH percentage change in mean heart dose.
- Maximum heart in the field on the FB plan with DIBH dose change to the heart.

All *P*‐values were two‐tailed and *P* < 0.05 considered as significant. All analyses were performed using the R statistical software (R Core Team 2018, Vienna, Austria).

## Results

### Patients

Between March 2016 and April 2017, 41 patients were recruited to the wider project of which this was a part. No right‐sided patients with internal mammary nodes presented during the time of recruitment. Of these, 31 patients were analysed in this study. The data of one patient, where it was decided that there was no dosimetric benefit observed with DIBH, has been included in the analysis as both a FB and DIBH plan had been generated before the decision was made to treat in FB. Patient demographic and clinical information are displayed in Table 3.

**Table 3:** Patient demographic and clinical history

Characteristics	Value (*N* = 31)
Age in years (mean, SD, range)	54.1 (SD 9.1, range 34–70)
BMI (mean, SD, range)	25.1 (SD 5.6, range 18.4–40.5)
*Primary site (%)*	
Left breast unspecified	20 (65%)
Left upper outer quadrant of breast	7 (23%)
Left upper inner quadrant of breast	2 (6%)
Left lower inner quadrant of breast	2 (6%)
*T stage (%)*	
1	23 (74%)
2	6 (19%)
3	2 (7%)
*N stage (%)*	
0	25 (81%)
1	6 (19%)
*Prescription dose (%)*	
42.5 Gy in 16#	18 (58%)
50 Gy in 25#	13 (42%)
Smokers (%)	6 (19%)
*Comorbidities (%)*	
Diabetes	3 (10%)
Cardiac disease	2 (6%)
Hypertension	6 (19%)

BMI, body mass index.

### DIBH versus FB plans

The median difference in patient separation between the FB and DIBH plans was −0.1cm (IQR − 3.2 to 2.6 cm). Dose reductions to the heart and LAD are displayed in Table 4. Of the 31 patients, 90.3% had a reduction in maximum heart dose, 87.1% a reduction in maximum LAD dose and 96.8% a reduction in mean heart dose. Plans were generated following the objectives outlined in Table 1 and PTV coverage was comparable between the FB and DIBH plans with a mean PTV V95%_Reference dose_ of 94.9% and 94.4%, respectively.

**Table 4:** Heart max, LAD max and mean heart dose reductions

Reduction in dose between FB and DIBH	Median (IQR)	Range
Max heart dose (Gy)	15.84 [4.73–28.64]	−2.16 to 39.24
Max LAD dose (Gy)	14.08 [4.68–33.77]	−3.54 to 44.96
Mean heart dose (Gy)	1.02 [0.26–1.73]	−0.14 to 6.51
42.5 Gy PD	0.53 [0.2–1.35]	−0.14 to 2.14
50Gy PD	1.62 [1.02–3.16]	0.03 to 6.51

### Relationship analysis

Patient breath hold duration times were similar ranging from 22 s to 25 s. There was no significant difference (*P *= 0.32) between the ABC device lung threshold value between patients who were non‐smokers and who were smokers, with average of 1.5 L (±0.3 L) and 1.6 L (±0.3 L) respectively. Also, there was no significant relationship between age and lung threshold value (*P* = 0.2). No correlations were found between heart, LAD and ipsilateral lung dose with either lung threshold value or patient separation. However, correlations were found with BMI, PTV volume, lung volume change and the maximum heart in the field.

#### Body Mass Index

**Figure 1:**
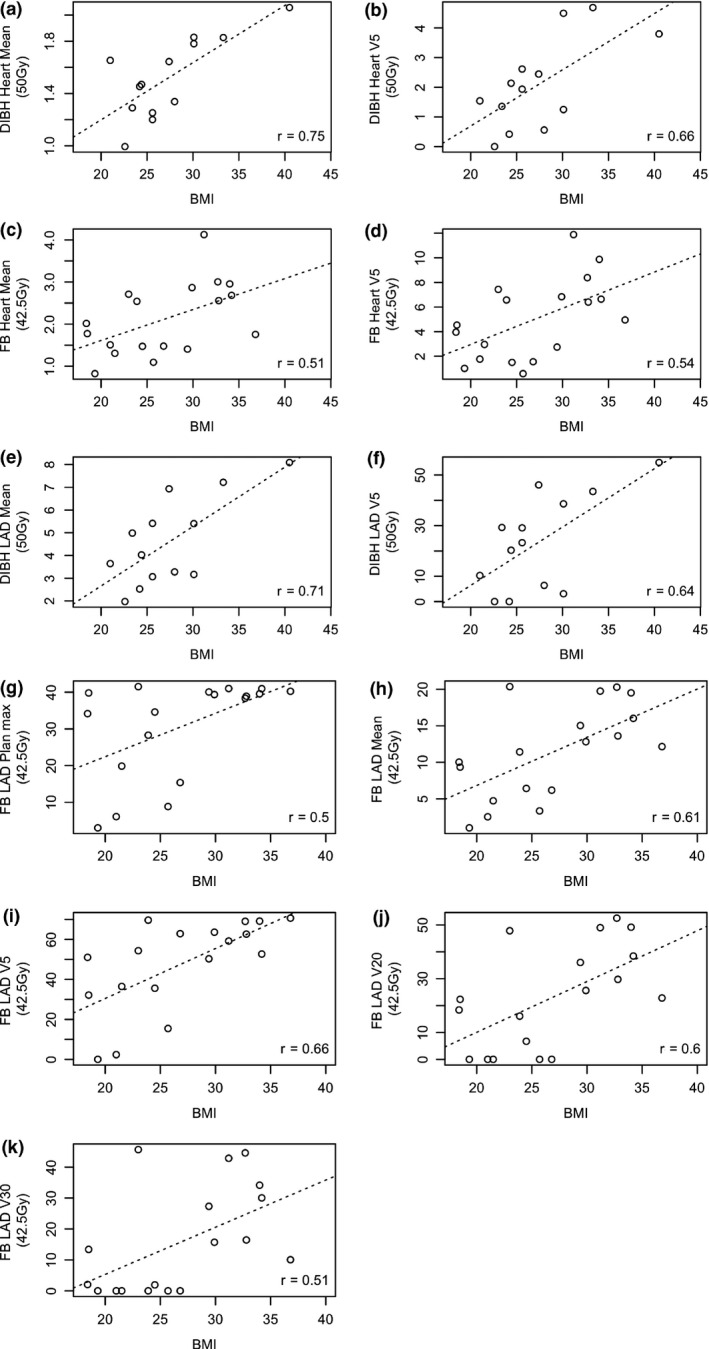
Correlation plots between BMI and DIBH heart and LAD doses. FB – free breathing, DIBH – deep inspiration breath hold, BMI – body mass index, LAD – left anterior descending artery

A significant positive relationship was found between BMI and the DIBH heart mean and V5 doses (Fig. 1a,b), BMI and FB heart mean (*P* = 0.03) and V5 doses (Fig. 1c,d), and between BMI and DIBH LAD mean and V5 (Fig. 1e,f). This showed that as BMI increased, the mean and V5 heart doses also increased. A moderate significant positive correlation was found between BMI and LAD plan max, mean, V5, V20 and V30 for the FB group. (Fig. 1g,k).

#### Change in PTV volume

**Figure 2:**
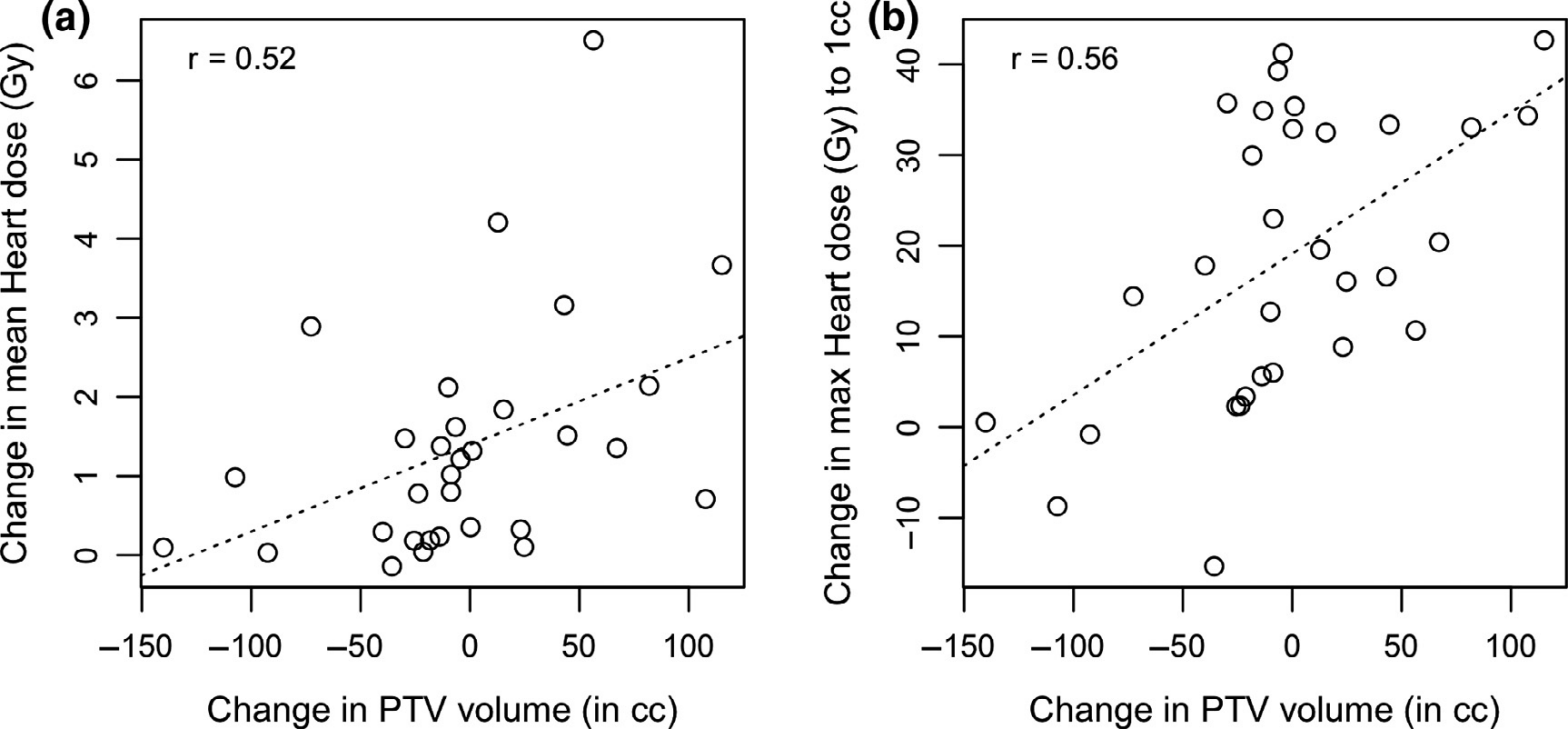
Correlation plots for FB to DIBH change in PTV volume and change in heart doses

Thirteen patients (41.9%) showed a reduction in PTV volume from FB to DIBH and 18 patients (58.1%) had an increase in PTV volume. The mean difference in PTV volume between FB and DIBH was 2.6cc (± −56.5cc). A significant moderate positive correlation was found between the change in PTV volume and the change in mean and maximum heart dose to 1cc (*r* = 0.52, *P* = 0.003 and 0.56, *P* = 0.001 respectively) from FB to DIBH (Fig. 2a,b). As the change in PTV volume increased, the overall mean heart dose reduction between FB and DIBH and the maximum heart dose to 1cc increased.

#### Change in lung volume

**Figure 3:**
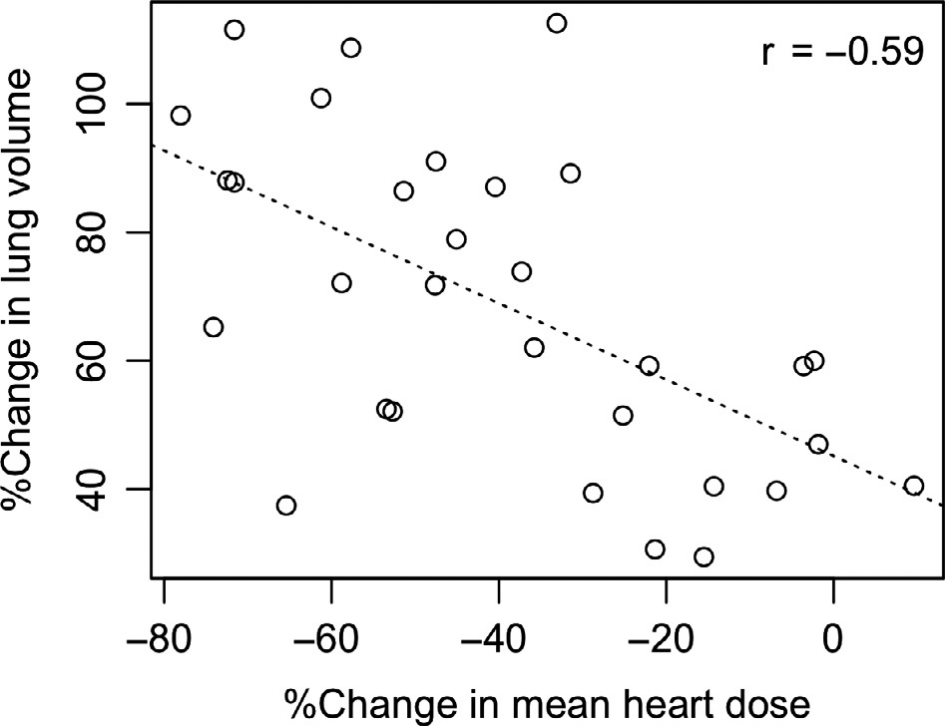
Correlation plots for FB to DIBH percentage change in mean heart dose and lung volume

The change in lung volume increased by a mean of 68.5% (± 24.9%) between FB to DIBH. A significant negative relationship was found between percentage change in lung volume and percentage change in mean heart dose (average of −39.3% ± 24.6%) from FB to DIBH (*r *= −0.59, *P* < 0.001) (Fig. 3), indicating that as the percentage change in lung volume decreased, the percentage change in mean heart dose also decreased.

#### Maximum heart in field

**Figure 4:**
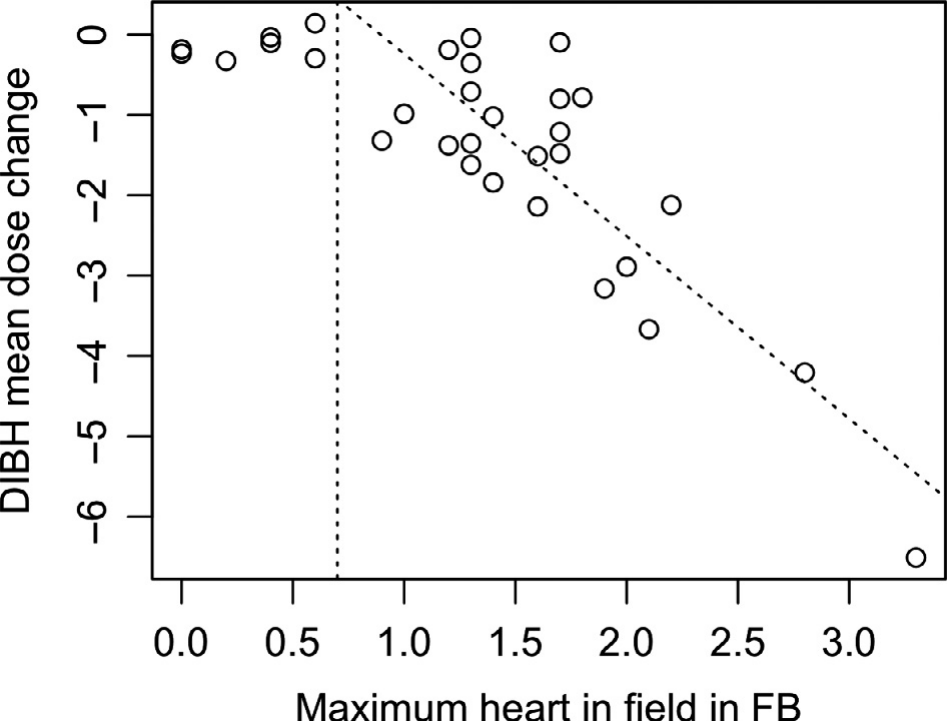
Correlation plot between FB maximum heart in field and FB to DIBH mean heart dose reduction

A significant difference in the maximum heart in field between DIBH and FB was found (*P* < 0.001). There was less heart in the field with DIBH when compared to FB with a mean difference of − 1 cm ± 0.7 cm (range = −2.4 to 0.1). A strong significant negative correlation was found between the maximum heart in field in FB and the DIBH mean heart dose change (*r* = −0.73, *P* < 0.001). As the amount of heart in the field in FB increased, the amount of dose to the heart also increased. On further investigation it was found that the relationship between these variables seemed to be almost non‐existent for a maximum heart in field value less than 0.7cm and became stronger with values greater than 0.7cm (*N* = 24, *r* = −0.81) (Fig. 4). Those results would need to be investigated on a larger cohort of patients due to the large imbalance in numbers between these two groups.

## Discussion

This exploratory study aimed to detect potential factors associated with a reduction in heart dose that could identify patients that may benefit most from the use of DIBH. Many factors were assessed, both those that could be evaluated prior to and after CT simulation for RT. However, only factors that could be evaluated after CT simulation were found to have a significant relationship with reduction in heart dose. Moderate to strong correlations were found between BMI, changes in PTV and lung volume and heart dose when DIBH was employed however no thresholds were discovered that could be used to select appropriate patients. The only possible threshold identified was the maximum heart in the field in FB with those patients with greater than 0.7 cm heart in the field experiencing greater reductions in mean heart dose. This association will require further investigations on a larger cohort of patients. With the constant growth in demand for DIBH and the resource intensive nature of the technique, knowledge of factors that could identify breast cancer patients who may benefit most from DIBH would be helpful. DIBH involves an increased workload for clinical staff, potential increases in treatment time and increased drain on departmental resources due to the extra equipment required.^7^ Additionally, it has been reported that DIBH can place extra strain on the patient potentially resulting in higher levels of fatigue and anxiety.^19^ The identification of potential predictive factors would therefore be advantageous to both patients and departments.

The dosimetric reductions achieved with DIBH in this study were comparable to dosimetric reductions reported in the literature. This study found that DIBH resulted in a median reduction in heart mean dose of 1.3–2.1 Gy, maximum heart dose of 15.8Gy and maximum LAD dose of 14.1 Gy. Similarly, Bruzzaniti et al^20^ found that the maximum doses to the heart and LAD and the mean heart dose were significantly lower in DIBH, (minimum 78.3% and 2.6% decreases with respect to FB, respectively) regardless of the prescription. Remouchamps et al^21^ found that moderate DIBH significantly reduced heart doses reporting a mean reduction of 81% in patients receiving locoregional breast irradiation using a mono‐isocentric 3‐field technique. Hayden and colleagues^22^ reported that using DIBH resulted in significantly larger total PTV volumes and smaller maximum heart depth and irradiation volume compared to FB scans. Interestingly this study found that 41.9% of patients had a reduction in PTV volume from FB to DIBH, with 58.1% having an increase in PTV volume from FB to BH. The mean difference was small however at 2.56cc.^22^

A handful of studies have attempted to identify potential predictive factors for the use of DIBH. Register and colleagues evaluated 64 breast cancer patients, comparing DIBH and FB plans. Regression analysis found that only a change in heart volume in field (HVIF) independently predicted for cardiac sparing.^12^ However, as HVIF relies on numerous factors such as volume of deep inspiration and the amount of contoured heart tissue exposed to radiation, it cannot be predicted by measuring a specific anatomic change between image sets. They concluded that they could not identify a fast and easily measurable predictive surrogate. Rochet et al^13^, when analysing predictors of cardiac exposure, found that free breathing cardiac contact distance is potentially a good predictor for cardiac exposure without compromising target coverage. They reported that a longer cardiac contact distance, the higher the heart dose. Lin and colleagues^10^ investigated a variety of anatomical factors in 16 patients including breast and heart volumes and heart in field and reported that the development of a predictive model is challenging for these patients. Similarly, Ledsom et al^9^ and Czeremszynska et al^6^ could not identify specific characteristics or thresholds that could accurately identify patients who would benefit most from DIBH despite finding correlations between patient related and dosimetric parameters.

In this present study, despite discovering correlations between reductions in heart and LAD dose and BMI and changes in PTV and lung volumes, no threshold was able to be determined that could be used to predict which patients will benefit most. The maximum heart in field in FB was the only parameter where an indication of a potential threshold was found. A strong correlation with reduction in mean heart dose only existed when the maximum heart in field at FB was 0.7 cm or greater. This would suggest that a maximum heart in field in FB of ≥ 0.7 cm could be used to potentially identify patients who may benefit from DIBH. However, this needs to be validated in a larger patient cohort. It should be noted that, despite variations in the actual measurement taken, the majority of studies that have suggested potential predictive factors have identified these as being related to the amount of heart in the field at FB indicating this area warrants further investigation.^6^, ^12^, ^13^, ^14^ Although this is a factor that would most often be assessed after CT simulation, if a patient had diagnostic CT scans performed during the diagnosis phase, the radiation oncologist may be able to use these to assess the potential amount of heart in treatment field. This information could inform their decision regarding whether the patient would benefit from DIBH prior to CT simulation.

The strength of this study findings is limited by the number of patients investigated however, this was designed as an exploratory study to identify potential factors warranting further in‐depth investigation that could be used to select breast cancer patients who may benefit most from DIBH. As such, a larger cohort of patients is required to test the results.

## Conclusion

As DIBH is time and resource intensive from a departmental perspective and potentially increases fatigue and anxiety for patients, there is a need to develop a predictive profile to inform appropriate breast cancer patient selection for this technique. No significant relationships were found in this exploratory study. However, patients with greater than 0.7 cm maximum heart in the field in FB experienced greater reductions in mean heart dose than patients lower than 0.7 cm. This threshold for maximum heart in the field in FB warrants investigation in a larger patient cohort to test its validity.

## Conflict of Interest

No conflicts exist.
